# Association of Serum Uric Acid Levels in Meige’s Syndrome

**DOI:** 10.3389/fnins.2021.755056

**Published:** 2021-10-01

**Authors:** Haochen Guan, Zhi Geng, Weijie Yuan, Bowen Chang

**Affiliations:** ^1^Department of Nephrology, Shanghai General Hospital, Shanghai Jiao Tong University School of Medicine, Shanghai, China; ^2^Department of Neurology, Second People’s Hospital of Hefei City, The Hefei Affiliated Hospital of Anhui Medical University, Hefei, China; ^3^Division of Life Sciences and Medicine, Department of Neurosurgery, The First Affiliated Hospital of USTC, University of Science and Technology of China, Hefei, China

**Keywords:** Meige’s syndrome, uric acid, antioxidants, movement disorders, Burke-Fahn-Marsden dystonia rating scale score

## Abstract

Uric acid (URIC) is a natural antioxidant, and it has been shown that low levels of URIC could be a risk factor for the development of Parkinson’s disease. Our aim was to investigate whether URIC also plays a role in Meige’s syndrome (MS). We conducted a cohort study to compare serum URIC levels between patients with MS and healthy controls. In addition, we analyzed the impact of URIC on the risk of MS and symptom severity. Compared with normal subjects, URIC content was remarkably decreased in MS patients. In addition, URIC was regarded as a protective factor for MS, as verified by multivariate logistic regression models. We also found non-linear relationships between the levels of serum URIC and the incidence rate of MS and the Burke-Fahn-Marsden dystonia rating scale score. Our study is the first to show a connection between serum URIC levels and MS. Low serum URIC levels indicate an increased risk of MS incidence and more severe clinical symptoms. Our findings provide new insights into the prevention and treatment of MS.

## Introduction

Meige’s syndrome (MS) is a dystonia characterized by bilateral eyelid and involuntary facial muscle movement (Greene et al., 1995; LeDoux, 2009). The incidence is approximately 100 cases per 100,000 people (Defazio et al., 2004). MS is currently considered movement disorder that commonly occurs in elderly female patients (Pandey and Sharma, 2017). Although the pathogenesis of movement disorder such as MS and Parkinson’s disease (PD) remains unknown, free radical accumulation (such as reactive oxygen species) and decreased antioxidants within brain tissue may be possible mechanisms (Savitt et al., 2006; Poewe et al., 2017).

Serum uric acid (URIC) has been identified as a natural antioxidant in the human body (Stocker et al., 1987; Becker et al., 1991). Recent meta-analyses and controlled studies suggest that reduced levels of URIC are related to PD (Jesus et al., 2013; Wen et al., 2017). In addition, two prospective articles demonstrated that people showing increased URIC contents may have a lower susceptibility to PD (Davis et al., 1996; Gao et al., 2016) and a slower rate of decline of neurological function (Ascherio et al., 2009). A postmortem study revealed that the URIC content within brain tissue (particularly in the striatal substantia nigra and cortex) was remarkably decreased in PD cases compared with normal controls (Church and Ward, 1994; McFarland et al., 2013). These results suggest that URIC may be related to PD progression, since its reduction within brain tissue indicates antioxidant insufficiency. Whether serum URIC has a similar effect on MS is unknown. At present, the etiology and pathogenic mechanisms of MS remain unclear. It has been suggested that diverse environmental and genetic factors, mitochondrial dysfunction, and other factors are related to the oxidative stress (OS) of neurons (Steele et al., 2014). This study focused on investigating the serum URIC content in MS and evaluating its impact on the development of MS.

## Materials and Methods

### Meige’s Syndrome Patients and Normal Subjects

This retrospective cross-sectional study collected medical records from MS cases diagnosed at the Shanghai General Hospital and Hefei Second People’s Hospital between January 2018 and February 2021. Patients meeting the following criteria were included: those with MS, the main manifestations were double blepharospasm, oral and mandibular dystonia, and involuntary movements like facial dystonia; those not receiving treatment such as opioids or non-steroidal anti-inflammatory drugs; those without hematological disorders, hyperpyrexia, concurrent infectious disease, severe heart disease, metabolic disorder, inflammatory disease or medication for inflammatory disease, autoimmune disease, severe liver/kidney disease, other malignancies; and those with sufficient data on the biochemical index of fasting blood. In addition, this study also collected medical records of age- and sex-matched healthy subjects who underwent physical examinations at the same hospital. None of the healthy control subjects took any drugs to raise or lower uric acid. Our study protocols were approved by the Institutional Ethics Committee of the hospital. All participants in this study provided informed consent.

### Data Extraction

Patient clinicopathological and demographic variables, including sex, age, BFMDRS (Burke-Fahn-Marsden dystonia rating scale) score, and history of hypertension were collected from medical records. In addition, blood samples were collected upon admission to conduct kidney and liver tests, which were part of the standard workup. Each specimen was assayed by the Department of Clinical Laboratory 2 h post-collection. Specifically, URIC, albumin (ALB), blood urea nitrogen (BUN), and creatinine (CREA) levels were examined to assess kidney and liver function. The above clinical variables were determined using standard automatic counters. The epidemiological data, clinical evaluation, and laboratory tests were extracted from the same visit of each patient.

### Statistical Analyses

R^1^ and Empower (R)^2^ (X&Y solutions, Inc., Boston, MA, United States) software were used for statistical analysis. First, the normal distribution of variables was assessed using the Kolmogorov–Smirnov test, and normally distributed data were assessed by one-way analysis of variance or two-tailed Student’s *t*-test. Simultaneously, non-parametric data were compared across diverse groups using the Mann–Whitney *U* test. A multiple logistic regression model was employed to evaluate the relationships between inflammatory markers and MS. Additionally, the value of the area under the receiver operating characteristic (ROC) curve (AUC) was determined to assess the significance of URIC in the diagnosis of MS. The two-piecewise linear regression model was also utilized to examine the role of URIC in predicting MS and BFMDRS scores using the smoothing function. Typically, trial and error were utilized to determine the threshold level (turning point), such as selecting the turning point down a preset interval and later selecting a turning point giving the maximum model likelihood. After *post hoc* analysis, we determined the outlier by adopting the value with maximal specificity and sensitivity. Statistical significance was set at *P* < 0.05.

## Results

### Study Participants

Altogether, data for 80 MS cases and 133 healthy controls were collected for final analyses. Table 1 shows the demographic data of all study participants. There were 29 (36.2%) male and 51 (63.7%) female MS patients, with ages ranging between 39 and 81 years. There were 72 (54.1%) male controls, aged between 29 and 86 years.

**TABLE 1:** Characteristics of the patients with Meige’s syndrome and healthy controls.

Variables	Healthy control	Meige’s syndrome	*P*-value
No.	133	80	
Gender			0.011
Male	72 (54.1%)	29 (36.2%)	
Female	61 (45.9%)	51 (63.7%)	
Age	55.5 ± 17.9	54.2 ± 10.1	0.547
Uric acid	317.2 ± 71.0	250.5 ± 66.9	< 0.001
CREA	65.2 ± 20.9	62.4 ± 13.9	0.283
BUN	5.3 ± 1.4	5.9 ± 1.5	0.013
ALB	42.0 ± 3.7	42.3 ± 4.1	0.698
Hypertension			0.109
No	94 (70.7%)	48 (60.0%)	
Yes	39 (29.3%)	32 (40.0%)	

### Comparing the Biochemical Indexes Between Meige’s Syndrome Patients and Normal Subjects

Table 1 shows the features of all study participants. Differences in ALB, BUN, CREA, and diabetes history were not significant between the two groups. In contrast, URIC levels of MS cases (250.5 ± 66.9 μmol/L) decreased significantly in comparison with healthy controls (317.2 ± 71.0 μmol/L; *P* < 0.05).

### Relationship of Uric Acid With Meige’s Syndrome

**FIGURE 1:**
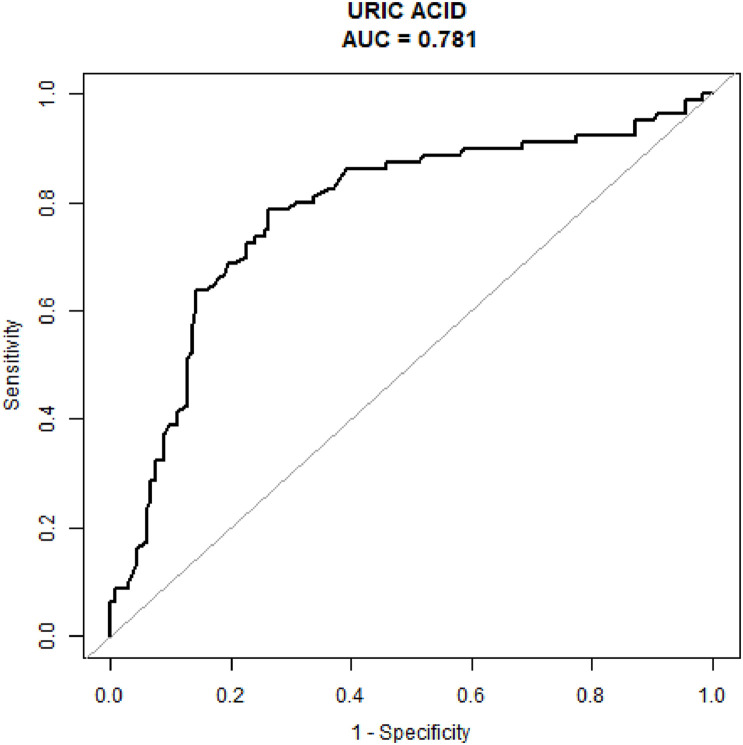
The diagnostic value of serum uric acid levels was evaluated using receiver operating characteristic analysis testing patients with Meige’s syndrome against healthy controls.

Table 2 displays the relationships between MS and diverse variables, such as sex, age, ALB, BUN, CREA, URIC, and hypertension history. URIC content was closely related to MS, and increased URIC content served as a protective factor for MS. Table 3 presents the above relationships assessed through multivariate analyses. As suggested by multivariate analysis, URIC content was closely related to MS (Odds ratio [OR] = 0.985; 95% confidence interval [CI]: 0.980–0.990; *P* < 0.001). After adjusting for confounders such as sex, age, and hypertension history, the results remained largely unchanged, which confirmed that the decreased URIC content served as a risk factor for CH (OR = 0.984; 95% CI: 0.979–0.990; *P* < 0.001). The value of URIC in diagnosing MS patients (ROC curves) is shown in Figure 1. Our results indicated that the URIC values performed the best in diagnosis, with an AUC value of 0.781 and the specificity and sensitivity were 0.7368 and 0.7875, respectively (Figure 1).

**TABLE 2:** Association between each variable and Meige’s syndrome.

Variables	Statistics	Meige’s syndrome OR (95% CI)	*P*-value
Gender			0.011
Male	101 (47.418%)	1.0	
Female	112 (52.582%)	2.076 (1.175, 3.668)	
Age	55.047 ± 15.397	0.994 (0.978, 1.011)	0.502
Uric acid	292.129 ± 76.528	0.985 (0.978, 0.992)	<0.001
CREA	64.120 ± 18.626	0.991 (0.977, 1.005)	0.223
BUN	5.531 ± 1.492	1.266 (1.050, 1.526)	0.013
ALB	42.119 ± 3.875	1.014 (0.942, 1.093)	0.706
Hypertension			0.111
No	142 (66.667%)	1.0	
Yes	71 (33.333%)	1.607 (0.897, 2.877)	

**TABLE 3:** Multivariate regression for effect of serum uric acid levels on Meige’s syndrome.

Variable	Non-adjusted		Model I		Model II	
Uric acid	OR (95% CI)	*P*-value	OR (95% CI)	*P*-value	OR (95% CI)	*P*-value
	0.985 (0.980, 0.990)	<0.001	0.985 (0.980, 0.991)	<0.001	0.984 (0.979, 0.990)	< 0.001

*Model I adjusted for age and gender. Model II adjusted for age, gender, history of hypertension. CI, confidence interval; OR, odds ratio.*

**FIGURE 2:**
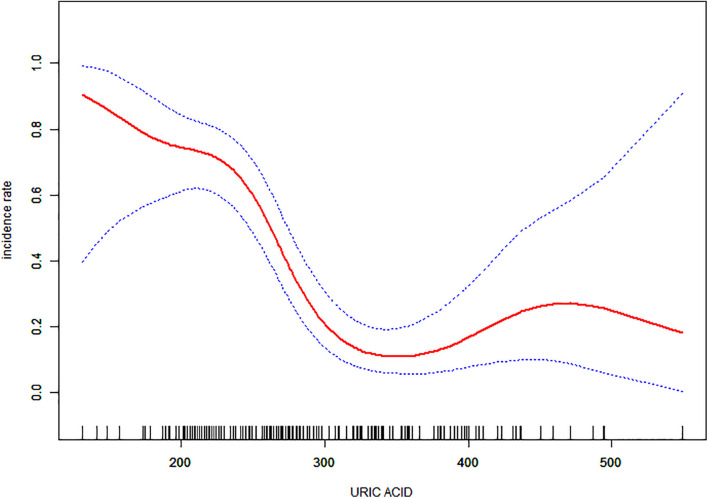
The association of serum uric acid levels between the incidence rate of Meige’s syndrome. The adjusted data for the incidence rate of Meige’s syndrome is plotted against serum uric acid levels with a curve indicating the shaped relationship between the two. A threshold serum uric acid level of 337 μmol/L existed for the regulation of uric acid.

**FIGURE 3:**
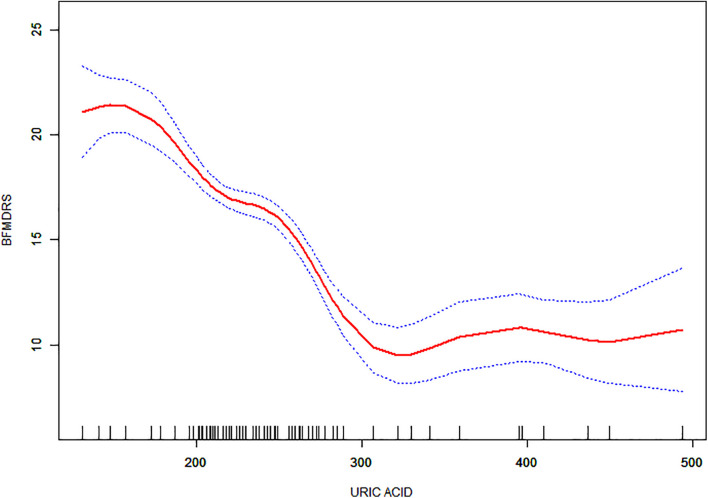
The association between serum uric acid levels and Burke-Fahn-Marsden dystonia rating scale scores. A non-linear relationship was observed, and a threshold serum uric acid of 330 μmol/L existed for the regulation of uric acid.

Non-linear relationships between serum URIC and the incidence rate of MS and the BFMDRS score were detected. The incidence rate of MS increased as serum URIC levels decreased to the turning point (URIC = 337 μmol/L). With a URIC level ≥ 337 μmol/L, the predicted dose–response curve conformed to the horizontal line (Table 4 and Figure 2). Similarly, the severity of symptoms increased with decreasing URIC levels to the turning point (URIC = 330 μmol/L). Likewise, the OR of the incidence rate of MS was 0.975 (95% CI: 0.968–0.983), and −0.065 (95% CI: −0.075 to −0.056) for BFMDRS scores (Table 5 and Figure 3).

**TABLE 4:** The threshold effect of serum uric acid levels on incidence rate of Meige’s syndrome assessments.

	OR (95% CI)	*P*-value
Uric acid <337 μmol/L	0.975 (0.968, 0.983)	<0.001
Uric acid ≥337 μmol/L	1.007 (0.997, 1.018)	0.186

*Adjusted for age, gender, history of hypertension. CI, confidence interval; OR, odds ratio.*

**TABLE 5:** The threshold effect of serum uric acid levels on score of BFMDRS assessments.

	OR (95% CI)	*P*-value
Uric acid <330 μmol/L	−0.065 (−0.075, −0.056)	<0.001
Uric acid ≥330 μmol/L	0.006 (−0.010, 0.021)	0.186

*Adjusted for age, gender, history of hypertension. CI, confidence interval; OR, odds ratio.*

## Discussion

Numerous studies have been conducted on the relationship between serum URIC levels and neurological diseases. Nonetheless, the association is still controversial because of the small sample sizes; besides, other confounders such as sex and age may have a certain impact. Currently, there are disputes regarding the role of URIC content in the prognosis prediction of neurological disorders. Increased URIC content has been suggested as a protective factor for functional results (Xue et al., 2017), whereas other studies suggest URIC content as a risk factor (Jung et al., 2018). The effect of URIC levels on MS has not been investigated until now.

This study analyzed URIC contents in MS patients and normal subjects. The results suggested that MS patients had decreased serum URIC content compared with normal subjects, suggesting that decreased URIC levels might serve as an MS-related risk factor. After adjusting for sex, age, and hypertension history, the heterogeneity remained significant. This result conforms to the opinion that URIC shows neuroprotection, which may be related to its role as an iron chelator and antioxidant (Davies et al., 1986). OS is related to numerous central nervous system (CNS) diseases, such as the dopaminergic cell decomposition of PD and early neuronal characteristics in Alzheimer’s disease (Moreira et al., 2006; Wei et al., 2018). Moreover, free radicals such as peroxynitrite may facilitate axonal demyelination and inflammation (Hooper et al., 2000). Therefore, the prevention of OS can delay the occurrence of such CNS diseases and improve their prognostic outcomes. URIC is a potent endogenous antioxidant that functions in resisting OS-mediated neuronal death and neurodegeneration, as suggested by *in vivo* and *in vitro* studies (Chen et al., 2013; Bartoli et al., 2017). As a result, the present work indicates that URIC exerts antioxidant and neuroprotective effects in MS.

URIC contents in MS cases are similar to those in PD cases, indicating a similar pathophysiological mechanism underlying neuronal injury. The neuroprotective effects of glutathione have been demonstrated in several studies (Fitzmaurice et al., 2003). The reduced glutathione content in PD is possibly due to the aberrant production, utilization, and catabolism of URIC and/or additional CNS antioxidants (Albers et al., 1999). Therefore, the decreased glutathione content in MS may be due to the decreased URIC content. Further studies are needed to explore this relationship.

Our study also found an interesting non-linear relationship between URIC levels, MS incidence and symptom severity, indicating that the incidence and severity of MS increased significantly when URIC was lower than 337 and 330 μmol/L. This suggests that monitoring and regulating URIC may be important for both patients with MS and healthy individuals. It is well known that the normal value of URIC is 149–416 μmol/L in males and 89–357 μmol/L in females (Maiuolo et al., 2016). When URIC is higher than normal, there is a risk of gout and cardiovascular disease (Jung et al., 2018). However, this study demonstrates that low serum URIC levels increase the risk of MS and the severity of symptoms in patients with MS. In summary, it is necessary to control serum URIC at an appropriate level. Therefore, our results suggest that controlling serum uric acid levels in the range of 337 μmol/L to the upper normal value is a reasonable goal. Thus, serum uric acid levels are a double-edged sword, and regulating URIC levels at an appropriate level is beneficial for preventing and delaying neurological diseases.

This study has some limitations. First, the MS cohort had a small sample size. Second, this study only extracted data regarding whether the study subjects had hypertension, but no information on detailed medication was gathered. Third, this study did not determine the long-term effects of URIC on MS.

The present study was the first to analyze the association of serum URIC levels with MS. Low serum URIC levels predict a higher MS risk and serious clinical symptoms. This study sheds light on future avenues of research for the prevention and treatment of MS.

## Data Availability Statement

The raw data supporting the conclusions of this article will be made available by the authors, without undue reservation.

## Ethics Statement

The studies involving human participants were reviewed and approved by Shanghai General Hospital Institutional Ethics Committee Second People’s Hospital of Hefei Institutional Ethics Committee. The patients/participants provided their written informed consent to participate in this study.

## Author Contributions

HG and ZG jointly completed the experiment and the writing. BC and WY took overall control of the whole study. All authors contributed to the article and approved the submitted version.

## Conflict of Interest

The authors declare that the research was conducted in the absence of any commercial or financial relationships that could be construed as a potential conflict of interest.

## Publisher’s Note

All claims expressed in this article are solely those of the authors and do not necessarily represent those of their affiliated organizations, or those of the publisher, the editors and the reviewers. Any product that may be evaluated in this article, or claim that may be made by its manufacturer, is not guaranteed or endorsed by the publisher.

## Footnotes

1
http://www.R-project.org

2
www.empowerstats.com

## Abbreviations

BFMDRS: Burke-Fahn-Marsden dystonia rating scale
MS: Meige’s syndrome
OS: oxidative stress
PD: Parkinson’s disease
URIC: uric acid.
